# Circadian disruption promotes tumor growth by anabolic host metabolism; experimental evidence in a rat model

**DOI:** 10.1186/s12885-017-3636-3

**Published:** 2017-09-06

**Authors:** Natalí N. Guerrero-Vargas, Raful Navarro-Espíndola, Mara A. Guzmán-Ruíz, María del Carmen Basualdo, Estefania Espitia-Bautista, Ana López-Bago, Ricardo Lascurain, Cinthya Córdoba-Manilla, Ruud M. Buijs, Carolina Escobar

**Affiliations:** 1 Departamento de Anatomía, Facultad de Medicina, UNAM, Universidad Nacional Autónoma de México, Ciudad Universitaria, 04510 México City, Mexico; 2 Departamento de Biología Celular y Fisiología, Instituto de Investigaciones Biomédicas, Universidad Nacional Autónoma de México, 04510 Mexico City, CP Mexico; 3 Departamento de Bioquímica, Facultad de Medicina, Universidad Nacional Autónoma de México, 04510 Mexico City, CP Mexico; 4 Departamento de Medicina experimental, Facultad de Medicina, Universidad Nacional Autónoma de México, 04510 Mexico City, Mexico

**Keywords:** Light at night, Circadian disruption, Tumor development, Inflammation, Metabolism and obesity

## Abstract

**Background:**

Light at night creates a conflicting signal to the biological clock and disrupts circadian physiology. In rodents, light at night increases the risk to develop mood disorders, overweight, disrupted energy metabolism, immune dysfunction and cancer. We hypothesized that constant light (LL) in rats may facilitate tumor growth via disrupted metabolism and increased inflammatory response in the host, inducing a propitious microenvironment for tumor cells.

**Methods:**

Male Wistar rats were exposed to LL or a regular light-dark cycle (LD) for 5 weeks. Body weight gain, food consumption, triglycerides and glucose blood levels were evaluated; a glucose tolerance test was also performed. Inflammation and sickness behavior were evaluated after the administration of intravenous lipopolysaccharide. Tumors were induced by subcutaneous inoculation of glioma cells (C6). In tumor-bearing rats, the metabolic state and immune cells infiltration to the tumor was investigated by using immunohistochemistry and flow cytometry. The mRNA expression of genes involved metabolic, growth, angiogenes and inflammatory pathways was measured in the tumor microenvironment by qPCR. Tumor growth was also evaluated in animals fed with a high sugar diet.

**Results:**

We found that LL induced overweight, high plasma triglycerides and glucose levels as well as reduced glucose clearance. In response to an LPS challenge, LL rats responded with higher pro-inflammatory cytokines and exacerbated sickness behavior. Tumor cell inoculation resulted in increased tumor volume in LL as compared with LD rats, associated with high blood glucose levels and decreased triglycerides levels in the host. More macrophages were recruited in the LL tumor and the microenvironment was characterized by upregulation of genes involved in lipogenesis (*Acaca, Fasn, and Ppar*γ), glucose uptake (*Glut-1)*, and tumor growth (*Vegfα, Myc, Ir)* suggesting that LL tumors rely on these processes in order to support their enhanced growth. Genes related with the inflammatory state in the tumor microenvironment were not different between LL and LD conditions. In rats fed a high caloric diet tumor growth was similar to LL conditions.

**Conclusions:**

Data indicates that circadian disruption by LL provides a favorable condition for tumor growth by promoting an anabolic metabolism in the host.

**Electronic supplementary material:**

The online version of this article (10.1186/s12885-017-3636-3) contains supplementary material, which is available to authorized users.

## Background

The alternation of day-night cycles is necessary for entrainment of the master circadian clock to efficiently transmit temporal signals to the organism in order to adapt behavioral and physiological responses to the cycling conditions of the environment [1]. Modern lifestyle, night-work and leisure schedules change the sleep-wake timing and due to the extended and sometimes inverted activity, individuals are exposed to light at night creating a conflicting signal to the circadian clock and disrupting circadian regulation of physiology.

Circadian disruption increases the risk to develop disease in humans [2] and rodents [3], it also promotes an obesogenic condition, altered metabolism [4–6], immune dysfunction [7, 8] and increased the vulnerability to develop cancer [9, 10]. In rodents, light at night increases the growth rate of mammary adenocarcinomas [11], chemical induced hepatocarcinogenesis [12], and accelerates aging and tumorigenesis in young rats [13]. These studies have related the increased tumor growth to the decreased nocturnal production of melatonin and its reduced blood concentration due to light at night. However, in addition to melatonin suppression, other deleterious changes triggered by constant illumination conditions (LL), may favor the process of tumor development.

Inflammatory environments and altered immune function are recognized as carcinogenic promoters [14–16]. Tumor-secreted inflammatory mediators such as Interleukin 6 (IL-6) and Tumor necrosis factor *α* (TNF-*α*), can regulate host metabolism in multiple tissues [17, 18], suggesting a possible role of an inflammatory state in mediating tumor-induced metabolic changes in the host. We have previously demonstrated that circadian disruption induces a increased inflammatory response [8] and promotes metabolic disturbances, including, dyslipidemia, insulin insensitivity and increased adipose mass [5], all of them leading to an obesogenic environment, which is an additional factor that could provide a favorable internal environment for tumor growth [19].

Here we hypothesized that circadian disruption induced by LL will favor tumor development via altering the inflammatory response and metabolism in the host, resulting in a propitious condition for the proliferative activities required for tumor growth.

## Methods

### Experimental design

The aim of this study was to investigate in rats exposed to LL and their controls the metabolic and the inflammatory state in the host, and the resulting conditions of the tumor microenvironment that may favor tumor’s growth. For this purpose, after 12 days of baseline, rats were randomly assigned to one of 2 groups: 1. Control LD, rats were left undisturbed in their home cages during 5 weeks and remained in 12:12 h LD; 2. Constant light (LL), rats were maintained with the lights on (200–250 lx at the level of the cage) for 5 weeks. Body weight and food intake were determined at the baseline and every week along the protocol. All animals included in the LL group were completely arrhythmic both in locomotor activity and body temperature after 5 weeks of LL exposure.

### Experiment 1. Behavioral and metabolic consequences of 5 weeks in LL

A first series of LD (*n* = 8) and LL (*n* = 8) rats were used to confirm arrythmicity of general activity and core body temperature (Tb) after 5 weeks in LL conditions. Intra-abdominal temperature sensors (iButtons) were implanted before starting experiments and programmed to measured Tb during the last two days on week 5 of the protocol. A glucose tolerance test (GTT) was performed at the end of the 4th week; glucose and triglycerides (TG) in plasma were assessed at the end of the 5th week.

### Experiment 2. Evaluation of the inflammatory response to LPS after 5 weeks in LL

A series of LD (*n* = 8) and LL (*n* = 8) rats were cannulated in the external jugular vein and were implanted with intra-abdominal temperature sensors (iButtons). After 1-week recovery (5 weeks in the lighting condition), rats received intravenous LPS (2 μg/kg) in the morning at ZT2 based on previous studies [8, 20] and in order to have the influence of light in both groups. Blood samples were collected from the jugular cannula and TNF-*α* was determined. In order to measure sickness behavior, food and fluid ingestion, body weight and temperature response were monitored before, following, 24 h and 48 h post the LPS administration. After this experiment, rats were euthanized and temperature sensors were collected for temperature analysis.

### Experiment 3. Evaluation of tumor development, tumor microenvironment and the influence of the tumor on the host metabolism

In another series of LD (*n* = 8) and LL (*n* = 8) rats, at the end of the 5th week, rats were subcutaneously inoculated with C6 tumor cells and 13 days later, rats were euthanized and tumors as well as blood were collected for further metabolic analysis of the host and tumor. Another series of LD (*n* = 8) and LL (*n* = 8) rats were subcutaneously inoculated with tumor cells and after 9 days a GTT was performed. All animals remained in their lighting schedules i.e., LD or LL until the end of the experiments.

### Animals and general housing conditions

Adult male Wistar rats weighing 190 to 200 g at the beginning of the experiments were obtained from the animal facility of the Faculty of Medicine of the Universidad Nacional Autónoma de México (UNAM). Animals were housed in individual cages placed in isolated lockers with controlled lighting conditions located in a soundproof monitoring room maintained at a controlled temperature of 22 ± 1 °C and with continuous air flow. All rats were given free access to food (Rodent Laboratory Chow 5001, Purina, Minnetanka, MN, USA) and water. For a baseline all rats were under a 12:12 h light-dark cycle (LD), lights-on at 7:00, defined as Zeitgeber time 0 (ZT0) and lights off at 19:00 h (ZT12).

### Automatic monitoring of general activity

General activity was automatically monitored daily with tilt sensors placed under the individual cages. Behavioral events were collected with a digital system (Omnialva SA de CV, México) and automatically stored every minute in a PC for further analysis. Analysis was performed with the program for PC SPAD9 designed for this system and based on Matlab. Double plotted actograms were constructed for each animal representing the number of activity counts every 15 min and periodicity with a χ^2^ periodogram for the last 14 days of the experimental protocol.

### Intra-jugular cannula insertion and intra-abdominal temperature sensors implantation

All surgeries were performed as previously described [20] using aseptic procedures.

For temperature recordings, the iButtons were programmed to collect core temperature data every 60 min and implanted in the rat peritoneum. For experiment 1, recordings started on week 5; for experiment 2, data were collected starting 2 days before LPS administration and continued until sacrifice. Temperature recordings were collected according to geographical time and the subjetive day-night phases for LL rats were selected based on the 12 h day and 12 h night of LD animals.

### Blood sample collection TNF-α and metabolic determinations

Blood samples (250ul) drawn from the intrajugular catheter were collected in Microvette®/500 tubes (Sarsted, Nümbrecht Germany) before LPS (0 min) and post-infusion times 40, 80, 120 and 180 min. Samples were centrifuged and plasma TNF-α levels were determined by ELISA according to the manufacturer’s recommendations (Invitrogen #KRC3011). Glucose and TG plasma levels were determined with enzymatic methods (ELITech Clinical Systems, France). Blood samples were taken from tail puncture between ZT2-ZT3 under ad libitum conditions.

### Glucose tolerance test

During week 4, the GTT was performed after 16 h of overnight fasting. A basal blood sample was obtained at ZT0 (7:00 h), and an intraperitoneal injection of 1 g of glucose/kg in saline solution was immediately given. After glucose administration, subsequent blood samples were collected from tail puncture (15, 30, 60 and 120 min respectively). Glucose level was determined with a blood glucose monitor (Glucose meter, Accu-Chek active. Roche).

### Inflammatory response

Inflammation was induced by a single intravenous (iv) injection of LPS (2μg/kg lipopolysaccharide from *Escherichia coli* serotype 0127:B8, Sigma-Aldrich, St. Louis, MO).

### Tumor xenografts

The glioma C6 cell line has shown to be a convenient model to assess factors influencing tumor proliferation. This C6 cell line has a similar growth rate in the brain and in the subcutaneous region, it is already visible on day 5 and it starts decreasing on day 15, providing a 10 day window for observations and manipulations. Moreover the histological characteristics are similar for C6 cells implanted in the brain and subcutaneously, showing high nuclear cell ratio, mitosis and pseudopalisading with small populations of GFAP positive cells [21]. Therefore subcutaneous implantation has the advantage that it can be measured with a caliper, and can be easily monitored externally without killing rats on different days. For this study the glioma C6 cell line was kindly provided by Dra. Patricia García López from the Instituto Nacional de Cancerología México and was obtained from ATCC® CCL-107™ (Rockville, Maryland, USA). This cell line was cloned from a rat glial tumor induced by N-nitrosomethylurea [22]. The cell culture was maintained as a monolayer in RPMI-1640 medium supplemented with 5% fetal bovine serum and incubated at 37 °C in a 5% CO_2_ atmosphere at high humidity. The C6 cell line was tested negative for *Mycoplasma*.

Rats were subcutaneously inoculated with 5 × 10^6^ C6-cells in the back right flank; tumor size was assessed every 2 days from day 7 to day 13. The volume of C6 tumors reaches a maximum on day 15 in intact rats, after which the tumor reabsorbs [21]. Tumor volume was determined with a caliper using the following relation: V = π/6 × (large diameter × [short diameter] ^2^).

### Tumor macrophages immunohistochemistry and cell count

Tumors were fixed in 4% paraformaldehyde (ph 7.2) for 24 h at 4 °C, and cryo-protected in 30% sucrose 1 mM PB (ph 7.2) for 3 to 4 days. Tumors were frozen and cut in 20 μm coronal sections at −20 °C. Free-floating tumor sections were incubated for 24 h under constant shaking at 4 °C with rabbit anti F4/80 antibody (1:2000; Santa Cruz) and were processed according to the avidin-biotin peroxidase method [20]. Immunoreactivity to F4/80 was quantified in six representative sections using a light microscope (Leica ICC50HD) and captured with a 40× ocular. Immunoreactive-positive areas were counted using computerized image analysis system (Image J, 1.42q, National Institutes of Health Bethesda, MD) using 12 squares grid over the tumor picture. Positive staining grids (inflammatory loci) were counted by free hand.

### Tumor q-PCR

Total RNA from tumors was harvested using Trizol reagent (life technologies). RNA was reverse transcribed to generate cDNA using SuperScript III first-stand synthesis super mix (Invitrogen). Specific primer sets (Additional file 1: Table S1) and Kapa Sybr Master Mix (Kapa biosystems) were used for qPCR. Data were collected using a A Prism 7000 real-time PCR system (Life Technologies), samples were run in duplicate. Relative quantification studies were performed with the collected data using the Prism 7000 System SDS software 1.3 (Life Technologies) and the relative expression ratio (R) of a target gene was calculated based on Efficiency and the CP deviation of an unknown sample versus a control, and expressed in comparison to the reference genes [23] hypoxanthine phosphoribosyltransferase (HPRT) and TATA box binding protein (TbP).

### Flow cytometry

Tumor dissociation was performed as previously described [24]. Cells were washed twice by PBS and counted in a Neubauer chamber; cell viability was evaluated using Trypan Blue dye exclusion. Immunofluorescence staining was carried out by antibodies to rat lymphocyte markers (Additional file 1: Table S2). In brief, 2 X 10^5^ cells were suspended in PBS containing 0.2% bovine serum albumin and 0.2% sodium azide, and incubated with fluorescent antibodies for 30 min at 8°C. After washing, 10,000 cells were analyzed on a MACSQuant flow cytometer (Miltenyi Biotech, Germany). First, acquired cells were gated by their physical properties (forward and side scatter); immediately, a second gate was done based on CD45 expression and forward scatter, from which was drawn a histogram to analyze CD43, CD3, CD4, CD8, CD45R and CD161 expression.

### Statistical analysis

Data are presented as mean ± standard error of the mean (SEM). Weight gain, tumor volume, core temperature, food and water intake, ad libitum-fasted glucose, glucose levels for the GTT and TNF-α plasma levels were compared with a two-way ANOVA for repeated measures for two factors (Condition LL or LD x time). Mean day-night temperature was compared with a two-way ANOVA. ANOVA’s were followed by Bonferroni’s post-hoc test for multiple comparisons. An unpaired one-tail Student T test was used to analyze food ingestion, serum TG, AUC, CD43 cells and genes measured in the tumor. Mann-Whitney test was used to analyze F4/80-IR positive grids.

All data and the Area Under the Curve (AUC) for plasma glucose were analyzed by using GraphPad Prism (version 6.03; Graph Pad Software, Inc.). Statistical significance was set at α = 0.05.

## Results

### Constant light induced loss of circadian rhythms in general activity and core body temperature

**Fig. 1:**
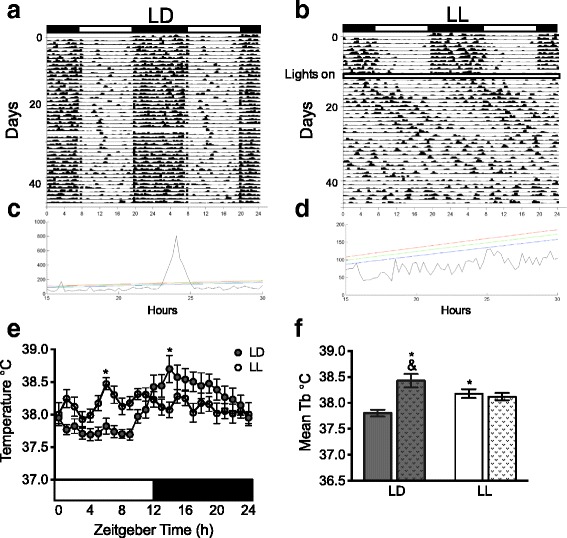
Constant light for 5 weeks disrupts circadian rhythms in general activity and core body temperature (Tb). (**a**) Representative double-plotted actograms from LD and (**b**) LL rats respectively. Black and white horizontal bars on top of the actograms represent night and day. The change in the lighting condition after 12 days of baseline (12:12 h LD cycle) is indicated with the legend “lights on” and the large white bar representing the constant light condition. (**c**) The χ^2^ periodogram test for the last 14 days demonstrates a 24 h rhythm for LD and (**d**) the absence of a circadian rhythm for LL rats. (**e**) Mean temperature values from the last 2 days of the lighting schedule for LD (grey circles) and LL (white circles) rats along 24 h. (**f**) Mean day-night temperature values for LD (grey bars) and LL (white bars), stripped bars in each group represent the night. Data are the mean ± SEM (*n =* 8/group). For E the repeated-measures two-way ANOVA, indicated significant interaction of the lighting condition versus time *p* < 0.0001. For F the two-way ANOVA indicated significant interaction of the lighting condition versus time *p* < 0.0001. The Bonferroni test indicated statistical difference LL from LD *p < 0.05 for F and indicated & *p* < 0.001 between day and night in the LD group

Control rats in LD exhibited a clear day-night general activity alternation, characterized by high activity levels during the night (Fig. 1a). In contrast, LL induced a progressively loss of general activity rhythmicity until no clear day–night difference was observed (Fig. 1b). The periodogram corresponding to the last 14 days of experiment confirmed circadian rhythmicity in general activity for all rats in LD (Fig. 1c) and loss of circadian rhythmicity for all LL rats (Fig. 1d).

Circadian rhythms in core body temperature (Tb) were also monitored during the last 2 days of the lighting protocol. LD rats showed a clear day-night Tb rhythm characterized by low temperature levels during the day and high levels during the night (Fig. 1e) while LL rats showed constant temperature values along the subjective day-night 24 h period. Interestingly, the mean daily temperature of LL rats was higher than the mean of the day values of LD rats (Fig. 1d; *p* < 0.05).

### Constant light modified metabolism in the host

**Fig. 2:**
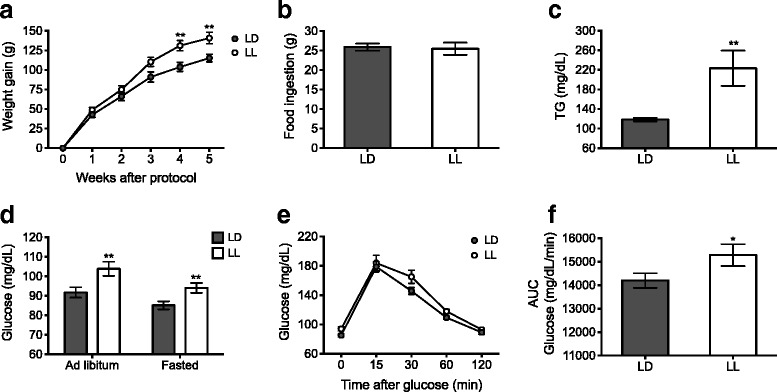
Constant light (LL) disrupts metabolism. (**a**) LL rats (white circles) gained more body weight along the 5-week protocol as compared with LD rats (grey circles). The repeated-measures two-way ANOVA indicated significant interaction of the lighting condition versus time, *p* = 0.0011. The Bonferroni test **p < 0.01 indicated statistical difference from LD. (**b**) Food ingestion assessed for 24 h during week 5 indicated no differences between groups. Data are expressed as mean ± SEM (*n =* 7–8/group). (**c**) Basal plasma TG and (**d**) glucose levels under ad libitum and fasted conditions were significantly increased in LL rats. Data are the mean ± SEM (*n =* 12/group), **p < 0.01 indicates statistical difference from LD; unpaired t test. (**e**) Glucose tolerance test (GTT, 0–120 min) and (**f**) area under the curve (AUC) following i.p. administration of 1 g of glucose/kg. Data are the means ± SEM (*n =* 14*–*15/group). * *p* < 0.05 indicates statistical difference from LD; unpaired t test

After 5 weeks, LL animals had gained more weight than the control LD rats; this reached significant difference from LD animals on weeks 4 and 5 of the protocol (Fig. 2a; *p* < 0.01). The increased body weight gain observed in LL rats, was not due to a difference in food consumption (Fig. 2b).

Plasma TG levels were higher in LL as compared with LD rats (Fig. 2c; *p* < 0.01); similarly, glucose plasma levels were significantly higher in LL rats as compared with LD rats both under fasted (before GTT) and ad libitum conditions (Fig. 2d; *p* < 0.01). In addition, LL rats showed an impaired glucose clearance, as demonstrated with the GTT (Fig. 2e-d; *p* < 0.05).

### Constant light increases the inflammatory response to LPS

**Fig. 3:**
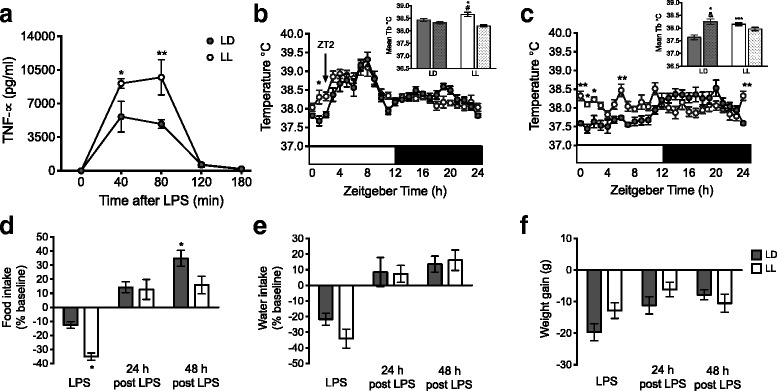
LL rats produced more TNF-α and showed increased sickness behavior in response to LPS. (**a**) TNF-α plasma levels after the administration of 2μg/kg of LPS. The repeated-measures two-way ANOVA indicated a significant effect due to the interaction condition versus time, *p* = 0.0065. The Bonferroni test **p < 0.01 indicated statistical difference from LD. (**b**) Thermoregulatory response to LPS. The arrow represents LPS administration at ZT2 for both groups. The mean day-night core body temperature (Tb) is shown in the box, for LD (grey bars) and LL (white bars), stripped bars in each group represent the night. The repeated-measures two-way ANOVA indicated a significant effect due to light condition *p* = 0.0107; and time *p* = 0.0011. The Bonferroni test *p* < 0.05 indicated statistical difference from LD and # between LL. (**c**) Thermoregulatory response 24 h post LPS. The mean day-night core body temperature (Tb) is shown in the box. The subjetive day-night phases for LL rats were selected based on the 12 h day and 12 h night of LD animals. The repeated-measures two-way ANOVA indicated effects due to the interaction condition versus time, *p* = 0.0011; the Bonferroni test indicated *p < 0.05 statistical difference from LD and & between LD. (**d**) Food intake, (**e**) Water intake and (**f**) Weight gain during the day of LPS administration, 24 and 48 h post LPS in LD and LL rats. Values are expressed as a percentage of the baseline value established prior to LPS administration. For d, the repeated-measures two-way ANOVA indicated effects due to lighting condition *p* = 0.0107 and time *p* = 0.0011. Data are the mean ± SEM (*n =* 6/group). *p < 0.05 indicates statistical difference from LD; with Bonferroni test

Basal TNF-α plasma levels, measured at time 0 were very low or undetectable in both LD and LL rats. LPS administration triggered a significant increase of TNF-*α* plasma levels in both groups, reaching the highest levels after 40 and 80 min, LL rats reached significantly higher TNF-α plasma levels as compared with LD rats (Fig. 3a; *p* < 0.05).

After the LPS challenge, analysis of Tb indicated that both groups exhibited an initial increase in Tb with a first peak 1 h after LPS administration and a second peak 5–6 h later, the mean temperature of the day in LL rats was significantly higher as compared to LD rats (Fig. 3b; *p* < 0.05) and the Tb difference between day and night in LD rats was dampened. 24 h post LPS injection Tb of both groups returned to pre injection levels (Fig. 3c). Both groups reduced food consumption on the day of LPS administration, this was more severe in LL rats, which consumed 41.66 ± 2.76% less food as compared to the 12.59 ± 2.41% reduction observed in LD rats (Fig. 3d; *p* < 0.05); water intake was also reduced in both groups (Fig. 3e). 24 h after LPS administration both groups increased food and water intake; nevertheless 48 h post LPS, LL rats were still consuming significantly less food than the LD group (Fig. 3d; *p* < 0.05).

This initial food and water reduction impacted on body weight for both groups on the day of LPS administration; however, there was no difference in the weight loss between groups (19 ± 2.67 g in LD and 12.83 ± 2.46 g in LL rats). Animals had not recovered body weight 48 h post LPS. Altogether these results indicate that LL aggravates the sickness response, especially cytokine production and food consumption.

### Inoculated tumor cells grow more in LL rats

**Fig. 4:**
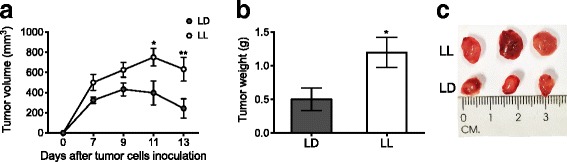
Constant light enhances tumor growth. (**a**) Tumor volume along 13 days after subcutaneous C6 cells inoculation. The repeated-measures two-way ANOVA indicated a significant interaction of condition versus time, *p* = 0.0240. The Bonferroni test indicated *p < 0.05, *p < 0.01 statistical difference from LD. (**b**) Tumor weight at day 13. *p < 0.05 indicates statistical difference from LD; unpaired t test. (**c**) Representative pictures from LD and LL tumors. Data are the mean ± SEM (*n =* 7/group)

Inoculated tumor cells formed bigger tumors in LL rats, that were significantly different from LD tumors on days 11 and 13 (Fig. 4a; *p* < 0.05). At the end of the experiment, isolated tumors from LL were also significantly heavier than LD tumors (Fig. 4b-c; *p* < 0.5). Together these findings suggest that LL induces a suitable environment for tumor growth.

### Tumor development changed the metabolic profile in the host

**Fig. 5:**
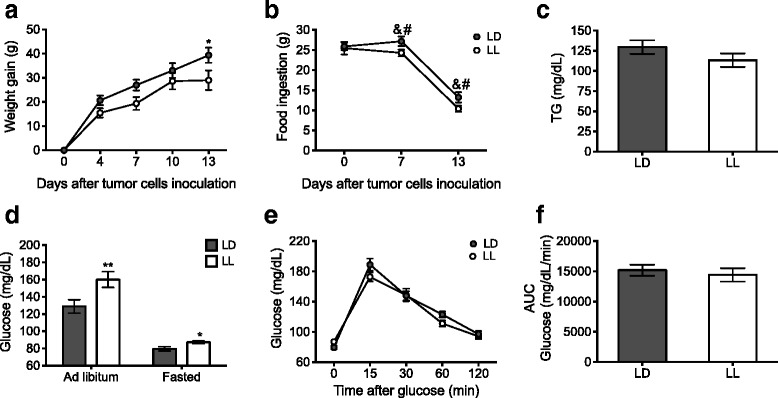
Tumor development modifies the metabolic profile of the host. (**a**) Accumulative gain weight along the 13 days after tumor cells inoculation (**b**) Food ingestion before, on day 7 and day 13 after tumor cells inoculation. Data are the mean ± SEM (*n =* 7/group). For A and B, the repeated-measures two-way ANOVA indicated a significant effect of time, *p* < 0.0001. Bonferroni test indicated *p < 0.01 statistical difference from LD, & between LD and # between LL. (**c**) TG and (**d**) glucose levels under ad libitum and fasted conditions in LD and LL tumor-bearing rats (13 days) D. Unpaired t test **p > 0.001 indicated statistical difference from LD. Data are the mean ± SEM (*n =* 7/group). (**e**) Glucose tolerance test (0–120 min) and (**F**) AUC following i.p. administration of 1 g glucose/kg. Data are the mean ± SEM (*n =* 8/group)

Tumor development affected the body weight between LL and LD groups (Fig. 2a). Before tumor inoculation LL rats were heavier than LD rats, 4 days after inoculation differences disappeared between groups and by the end of the experiment (13 days after) LD rats had gained more weight as compared to LL rats (Fig. 5a; *p* < 0.05), suggesting that in LL rats the increased tumor growth resulted in a higher metabolic demand.

Tumor development also decreased food ingestion in both groups as compared to their own basal levels (Fig. 5b; *p* < 0.05) without difference between groups. TG levels in LL tumor-bearing rats diminished 26% as compared to their previous condition. In contrast, TG levels in LD tumor-bearing rats increased 10% as compared to their previous condition; thus TG levels were not different between LL and LD tumor-bearing rats (Fig. 5c). The presence of the tumor induced an increase of glucose levels in both groups. LL animals increased 55.01% ± 6, while LD rats increased 45.92% ± 8 from their basal glucose levels. In addition, tumor development also increased fasting blood glucose levels in LL rats as compared to LD (Fig. 5d; *p* < 0.05); nevertheless glucose clearance was not different between LL and LD tumor-bearing rats on day 9 after tumor cells inoculation as demonstrated with the GTT.

In order to test whether the initial metabolic condition induced by LL may be the promoting factor for tumor growth, a different group of rats in LD condition, was exposed to a 1 h daily access to high sugar diet for 4 weeks (Additional file 2). The high sugar diet induced increased body weight and similar metabolic disturbances as observed in LL rats (Additional file 3: A-D). A sugar diet favored the growth of bigger tumors as compared to rats consuming a chow diet (Additional file 3: E; *p* < 0.01), reaching similar size as tumors in the LL rats at day 13.

### Tumors from LL rats recruit more macrophages

**Fig. 6:**
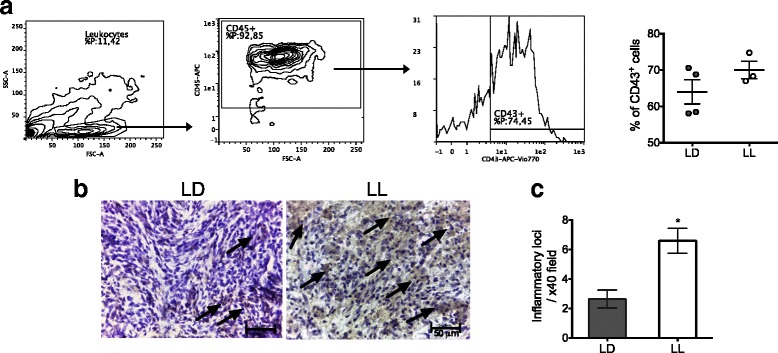
Tumors from LL rats recruit more macrophages. (**a**) Gating strategy employed to identify CD43^+^ cells in LD and LL tumors and percentage of CD43^+^ cells (*n* = 3–4/group). Analysis was done on whole lysates from tumors removed from LD and LL rats on day 13. (**b**) Immunohistochemistry staining of LD and LL tumors against an anti-macrophage antibody F4/80. Arrows indicate inflammatory loci. (**c**) Quantification of inflammatory loci in the grid area. Data are the mean ± SEM (*n* = 5–6/group) *p = 0.0173 indicates statistical difference from LD; Mann-Whitney test

Because the infiltration of immune cells is an important event that correlates with tumor growth or elimination (depending on the infiltrating immune cell type), we investigated the inflammatory condition in the tumor. Tumor infiltration of T cells (CD3^+^CD4^+^ and CD3^+^CD8^+^), NK cells (CD161^+^) and B cells (CD45R) was not different between LD and LL (Additional file 4: A-D). However, tumors of LL rats tend to recruited more monocytes (CD43^+^, Fig. 6a). Because monocytes are the precursors of macrophages, we evaluated the presence of macrophages inside the tumors, using the F4/80 marker. The inmmunohistochemical staining indicated that tumors from LL rats recruited more macrophages as compared to LD rats (Fig. 6b-c; *p* < 0.05). The increased number of tumor macrophages in LL did not result in increased levels of circulating TNF-α. Undetectable TNF-α plasma levels were measured on both LD and LL rats (Data not shown).

### The LL tumor microenvironment is characterized by an altered metabolic profile

In order to identify the factors in the tumor microenvironment that may favor its growth, a set of genes related with metabolism, cytokines, growth and angiogenesis pathways were evaluated in tumors obtained from LD and LL 13 days after inoculation.

**Fig. 7:**
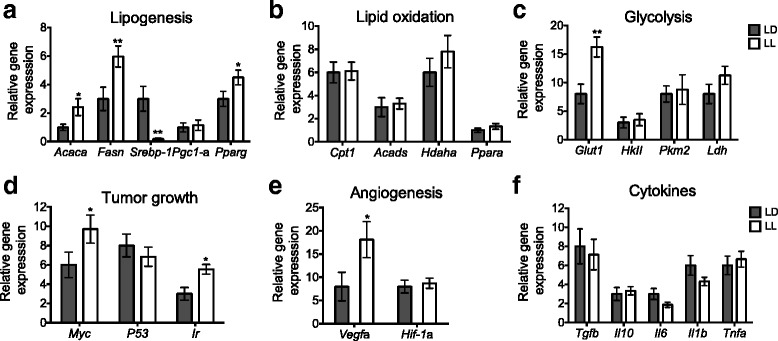
In tumors from rats exposed to LL the expression of genes involved in lipogenesis, glucose uptake, angiogenesis and cell proliferation was increased. (**a**) Relative expression of genes (*Acaca, Fasn, Srebp-1 and Ppar*γ) involved in lipogenesis; (**b**) Relative expression of genes involved in lipid oxidation (*Cpt1, Acads, Hadha and Pparα*); (**c**) glycolysis (*Glut-1*, *HkII*, *Pkm2*, *Ldh*); (**d**) Tumor growth (*Myc, P53, Ir*); (**e**) angiogenesis (*Vegfα, Hif1-α)* and (**F**) cytokines (*Tfgα, Il10, IL6, Il1α and Tnfα*). All genes were measured using qRT-PCR analysis. Gene expression in tumors was normalized to the expression of Hprt and Tbp as endogenous controls. Data are plotted as the mean ± SEM. (*n =* 7*–*10/group). *p < 0.05, *p < 0.01 indicates statistical difference from LD; unpaired t test

Genes involved in lipogenesis Acetyl-CoA carboxylase alpha (*Acaca*), Fatty acid synthase.

(*Fasn*) and Peroxisome proliferator activated receptor gamma (*Ppar*γ) were highly expressed in the tumors of LL rats as compared to LD tumors (Fig. 7a; *p* < 0.5), suggesting an up regulation of lipid production in tumors of LL animals in order to support their growth. Contrasting, Sterol regulatory element binding transcription factor 1 (*Srebp-1*) a transcriptional activator of the genes involved in lipogenesis was decreased in the tumors of LL rats (*p* < 0.05). The expression of genes related to lipid oxidation Carnitine palmitoyltransferase IA (*Cpt1a*), Acyl-CoA dehydrogenase (*Acads*), Hydroxyacyl-CoA dehydrogenase (*Hadha)* and Peroxisome proliferator-activated receptor alpha (*Ppar훼*) was not different between LD and LL tumors (Fig. 7b).

From the genes involved in glycolysis, the expression of the Glucose transporter 1 (*Glut1*) was increased in LL as compared to LD tumors (Fig. 7c; *p* < 0.05) suggesting a higher glucose uptake. The expression profile of other glycolytic genes Hexokinase II (*HkII)*, Pyruvate kinase muscle isozyme M2 (*Pkm2*) and Lactate dehydrogenase (*Ldh*) was not different between the two groups (Fig. 7c). In line with the increased lipogenesis and glucose transport, tumors from LL rats expressed high levels of the oncogene *Myc*, the Insulin transporter (*Ir*) and the pro-angiogenic gene *Vegf-α*(Fig. 7d and e; *p* < 0.05). In contrast, no differences between tumors in the two groups were found in the expression of the Hypoxia-inducible factor 1-alpha (*Hif1-α*) and the measured cytokines Transforming growth factor beta (*Tfgα*)*,* Interleukin 10 (*Il10),* Interleukin 6 (*IL6*), Interleukin 1 beta (*Il1α*) and *Tnfα* (Fig. 7f).

## Discussion

Light at night is a modern life style problem, especially for individuals living in big cities; it affects night workers as well as young people and children that are exposed to artificial light for extended hours of the night. The effects of light at night on human and rodent health have been the focus of several studies reporting a loss of body homeostasis, body weight gain, depression and increased tumor development [11, 25, 26]; however, the mechanisms involved in this process are not well established.

This study demonstrates that light at night disrupts the host’s metabolism as well as the inflammatory response creating an obesogenic environment, favorable for tumor growth. Tumors induced in LL rats showed an increased number of macrophages, expressed high mRNA levels of key enzymes involved in lipogenesis as well as in the uptake of glucose; this was associated with increased mRNA levels of markers of tumor development.

### LL disrupts metabolism

The control of cellular metabolism is essential for cell survival, and the role of aberrant cellular metabolism in cancer is becoming evident. In humans, over weight and an anabolic metabolism are associated with cancer development [19, 27]. Several systemic and metabolic alterations that accompany obesity, such as insulin resistance, hyperglycemia, fat accumulation, low-grade systemic inflammation and immune deregulation, also correlate with cancer development [28]. Present data are in agreement with this approach, since the increased tumor growth was associated with increased body weight gain, induced dyslipidemia, high glucose levels and altered glucose clearance in LL animals. Similar metabolic changes and tumor growth were observed after a high sugar diet, confirming that increase tumor development profits from the host’s metabolism shifted to an obesogenic condition. Indeed, high glucose levels are associated with poor survival in patients with glioblastoma [29, 30], the same kind of tumors induced in the present study after the inoculation of C6 cells in the rat [31]. Importantly, fasting regimens, which are associated with decreased levels of glucose and insulin, delayed the progression of cancer, have cancer preventive effects and increase the efficacy of chemotherapy agents [32–34].

It is well described that tumor cells “reprogram” the host’s metabolism in order to survive and proliferate under conditions that otherwise would arrest or kill normal cells [35]. We report that after 13 days of tumor induction, in both groups plasma glucose levels were increased while TG levels decreased in LL animals, correlating with increased tumor growth and suggesting TG uptake.

### Increased expression of glucose transporter 1 in LL tumors

Tumor cells take up nutrients such as glucose, lipids and aminoacids to fuel their metabolic pathways [36]. Glucose metabolism in cancer cells is known to be elevated due to altered membrane transport, that leads to increased intracellular glucose levels. Glucose is used by tumors to generate energy mainly through aerobic glycolysis (increased conversion of glucose to lactic acid to produce ATP) [37]. The main product lactate, is associated with increased tumor angiogenesis, heightened metastasis, and can also induce a pro-inflammatory state in the tumor microenvironment [38]. In line with this, tumors isolated from LL rats exhibited increased mRNA levels of the glucose transporter 1 (*Glut-1*), which promotes glucose import into the cytoplasm. Besides a primary substrate for ATP generation, glucose is a carbon source for the biosynthesis of other macromolecules; hence a critical nutrient for fast proliferating cells [39]. Contrasting, we did not find significant differences in the expression of key enzymes involved in the aerobic glycolytic pathway such as *HKII, Pkm2* and *Ldh*. Thus differences in enzymatic activity may be present since previous findings relate the growth of C6 tumor cells to the high expression of *Glut-1* coupled to glucose metabolism [40]. This is supported by observations in which glucose was withdrawn from culture medium inducing apoptosis in glioblastoma cell lines [41].

In this study the glucose tolerance test suggests insulin resistance induced by LL, which is in agreement with others findings [4], the increased *Ir* mRNA levels observed in LL tumors coupled with the increased insulin levels in LL observed by others, offer another possible pathway (46) by which tumor growth can be stimulated under the metabolic conditions of LL. This possible mechanism is further supported by the increased mRNA levels of the transcription factor *Myc* in LL tumors. Interestingly, besides regulating the transcription of genes involved in cell growth, cell proliferation, cell cycle, protein biosynthesis and apoptosis (under nutrient or growth factor deprivation conditions) [42], other genes targeted by the transcription factor *Myc* include key genes involved in glucose metabolism such as *Glut-1* [43], lipid metabolism and angiogenesis [44]. The increased expression of *Glut-1* probably promoting increased glucose influx to the LL tumor cells, together with the up-regulation of *Myc*, may favor glucose metabolism and the supply of acetyl-coA used as a substrate for lipid biosynthesis and for other nuclear processes.

### High lipid synthesis in LL tumors

Alterations in lipid metabolic pathways are another well-recognized metabolic adaptation that enables tumors to take up exogenous lipids or up-regulate endogenous synthesis (50,51). Decreased circulating TG levels in LL tumor bearing rats, suggest tumor lipid uptake, which is supported by the observed up-regulation of *Vegf-*α known target of *Ppar*-γ which was also up-regulated in LL tumors and it is known to be activated by fatty acids in the tumor microenvironment [45]. Moreover, present data suggest that LL tumors have increased lipid synthesis because they expressed high mRNA levels of all the key enzymes involved in lipid synthesis such as *Acaca* that generates malonyl-CoA from actetyl-Coa, *Fasn*, which catalyzes fatty acid chain elongation and *Ppar*γ, a transcription factor that regulates the expression of genes involved in lipid metabolism as well as tumorogenesis [46]. Strikingly, LL tumors expressed decreased mRNA levels of *Srepb-1* (a transcription factor that regulates the activation of genes involved in fatty acid synthesis), which suggest the role of other regulatory mechanisms for the increased expression of lipogenic genes in LL tumors. The increased fatty acids synthesis observed in LL tumors may favor energy production, cell signaling and tumor growth by inducing membrane synthesis, angiogenesis, migration and immunosuppression [46].

### Constant light disrupts the inflammatory response to LPS

Undetectable TNF-α plasma levels were measured in LL rats before the LPS challenge, indicating that LL increases the sensitivity to an immune challenge without changing the inflammatory state of the host at least in the circulation, as observed in other circadian desynchronization protocols such as experimental shift-work and jet lag in rodents [8, 47]. However, LL aggravated certain components of the sickness response such as cytokine production, and food consumption after LPS administration, which is in agreement with other studies [7]. Constant light also decreases the amplitude of the diurnal rhythmicity of leukocyte counts as well as the number and cytotoxicity of splenic NK cells in rats [48, 49]. Moreover, rats exposed to LL produce fewer antibodies in response to a T-cell dependent antigen [50]. Altogether these results also indicate that LL affects the function of the immune system in a way that may favor the development of disease and tumor growth.

### The inflammatory microenvironment of LL tumors

The exacerbated inflammatory response observed in LL animals suggested a deregulated inflammatory response affecting the tumor microenvironment. Our analysis confirmed that LL tumors recruited more macrophages as compared to LD tumors favoring tumor growth. Macrophages are the major immune cell population recruited in gliomas [51] and support tumor progression, angiogenesis, metastasis and immunosuppression [52]. In this sense, increased number of TAMs observed in LL tumors may have contributed to the observed tumor growth via the production of soluble factors such as VEGF a well-recognized angiogenic promoter. Here we show that the highly macrophage infiltrating LL tumors expressed increased pro-angiogenic factor *Vegf-a* mRNA levels, which regulates blood vessel formation but also exert mitogenic actions that may contribute to the enhance tumor growth observed in LL rats. Importantly, targeted deletion of TAMs in glioma xenografts promotes tumor regression [53].

Angiogenesis is an essential mechanism for tumor growth and maintenance, which may occur in response to environmental cues such as hypoxia stabilizing the transcription factor *Hif-1α,* that in turn activates the expression of angiogenic genes like *Vegf-a.* Levels of the *Hif-1α* mRNA were not different between LL and LD tumors, which can be explained by its relatively short-lived mRNA [54], or the oscillating O2 tumor levels (over the course of hours and days), which induce periodic fluctuations of tumor *Hif-1α* expression [55].

## Conclusions

The obesogenic metabolism observed in LL hosts associated to an altered immune response may have favored a propitious internal tumor environment. We have demonstrated that tumors from LL rats up-regulate key enzymes involved in glucose uptake and lipogenesis, which correlates with increased expression of tumor growth markers. Of clinical relevance is the fact that circadian disruption by LL exposure induces several metabolic features that are also observed in Type II diabetes mellitus patients or with metabolic syndrome; conditions that also are associated with increased cancer incidence.

Light at night suppresses melatonin in both, diurnal (humans) and nocturnal subjects [56–58] and has shown to exert adverse effects in diurnal species in a similar way as in nocturnal rodents [59, 60]. In this regard light at night is an environmental risk factor that appears to favor conditions for tumor growth, similar to obesity and diabetes. Present data highlight the importance of developing strategies to prevent circadian disruption and raise the need to continue exploring the link between circadian regulation and health problems including cancer.

### Limitations of our study

The tumor cell line used in this study does not enable us to follow tumor development at latter survival times because for this type of cells the host immune system induces tumor involution. However, this cell line allowed us to study tumor development in rats with an intact immune system and the interaction with the host’s homeostatic conditions. Although we induced the tumor by inoculating tumor cells, present data suggest that the metabolic condition observed in LL rats per se may promote spontaneous tumor formation at later stages as has been recently demonstrated in a model of circadian desynchronization by chronic jet lag exposure [61]. More studies are necessary to corroborate this.

## Additional files

Additional file 1: **Table S1.** and **Table S2.** (PDF 102 kb)

Additional file 2: Supplementary methods. (PDF 67 kb)

Additional file 3: High sugar diet (HS) induces a suitable metabolic environment for tumor growth. (A) HS rats (white circles) gained more body weight along the 4-week protocol as compared with chow diet rats (grey circles). Data are the mean ± SEM (*n =* 7/group). The repeated-measures two-way ANOVA indicated significant effects for condition versus time, interaction *p* = 0.0014. The Bonferroni test ***p < 0.001 indicated statistical difference from chow diet. (B) HS rats ingest more Kcal in 24 h. Data are the mean ± SEM (*n =* 7/group). ** *p* < 0.01 indicates statistical difference from chow diet; unpaired t test. (C) Basal plasma triglycerides (TG) and glucose levels (D) under ad libitum conditions were significantly increased in HS rats. Data are the mean ± SEM (*n =* 6–7/group), **p < 0.01, ***p < 0.001 indicates statistical difference from chow diet; unpaired t test. (E) Glucose tolerance test (GTT, 0–120 min) following i.p. administration of 1 g of glucose/kg. Values are expressed as mean ± SEM (*n =* 7/group). The repeated-measures two-way ANOVA indicated significant effects for condition versus time interaction *p* = 0.016. The Bonferroni test **p < 0.01 indicated statistical difference from chow diet. (F) Tumor volume along 13 days after subcutaneous C6 cells inoculation. The repeated-measures two-way ANOVA indicated a significant interaction for condition versus time, *p* = 0.0032. Data are expressed as mean ± SEM (*n =* 4-7group). The Bonferroni test indicated **p < 0.01 statistical difference from chow diet. (PDF 328 kb)

Additional file 4: Tumors from LL and LD rats similar percentages of immune cells. (A) Percentage of CD8^+^, (B) CD4^+^, (C) CD45R^+^ and (D) CD161^+^ cells. Data are expressed as the mean ± SEM (*n* = 3–4/group). Analysis was done on whole lysates from tumors removed from LD and LL rats on day 13. (PDF 23 kb)

## Abbreviations

*Acaca*: Acetyl-CoA carboxylase alpha
*Acads*: Acyl-CoA dehydrogenase
*Cpt1a*: Carnitine palmitoyltransferase IA
*Fasn*: Fatty acid synthase
*Glut1*: Glucose transporter 1
GTT: Glucose tolerance test
*Hadha*: Hydroxyacyl-CoA dehydrogenase
*Hif1-*α: Hypoxia-inducible factor 1-alpha
*HkII*: Hexokinase II
*Il10*: Interleukin 10
*Il1α*: Interleukin 1 beta
*Il6*: Interleukin 6
*Ir*: Insulin transporter
iv: Intravenous
LD: Light-dark cycle
*Ldh*: Lactate dehydrogenase
LL: Constant illumination conditions
LPS: Lipopolysaccharide
*Pkm2*: Pyruvate kinase muscle isozyme M2
*Pparα*: Peroxisome proliferator-activated receptor alpha
*Ppar*γ: Peroxisome proliferator activated receptor gamma
*Srebp-1*: Sterol regulatory element binding transcription factor 1
Tb: Core body temperature
*Tfgα*: Transforming growth factor beta
TG: Triglycerides
TNF-α: Tumor necrosis factor α
*Vegf*α: Vascular endothelial growth factor alpha
ZT: Zeitgeber time
