# Supplementary material for: Circadian disruption promotes tumor growth by anabolic host metabolism; experimental evidence in a rat model
Source: BMC Cancer. 2017 Sep 6;17:625. doi: 10.1186/s12885-017-3636-3 (PMC5585981; doi:10.1186/s12885-017-3636-3)
Supplement: Supplementary file 1 — Table S1. and Table S2. (PDF 102 kb) [file 12885_2017_3636_MOESM1_ESM.pdf]

Additional file 1.

Table 1. Primer sequences.

Gen	Reference number	Sequence	
Tnf α	XM008772775.1	Sense	TTCATCCGTTCTCTACCCAGC
		Antisense	GCCACTACTTCAGCGTCTCG
Il6	NM 012589.2	Sense	TAGTGTGCTATGCCTAAG
		Antisense	TATTGCCAGTTCTTCGTA
Il1β	NM031512.2	Sense	GGCAACCACTTACCTATT
		Antisense	ATTCACATATACAAGCAACAGA
Il10	NM012854.2	Sense	CCTCTGGATACAGCTGCGAC
		Antisense	GTAGATGCCGGGTGGTTCAA
Tgf β1	NM021578.2	Sense	AGAGATTCAAGTCAACTGTGGAG
		Antisense	CCAAGGTAACGCCAGGAA
Ppar α	NM013196.1	Sense	TGGAGTCCACGCATGTGAAG
		Antisense	CGCCAGCTTTAGCCGAATAG
Myc	NM012603.2	Sense	CACACAACGTCTTGGAACGTC
		Antisense	TCGCCGTTTCCTCAGTAAGT
Vegfα	NM001287114.1	Sense	ACGTTTTTGGGGAGCCTCAG
		Antisense	TGTCTTCTCTTTGCCAGGACC
Hif1-α	XM008764695.1	Sense	CAGGATTCCAGCAGACCCAG
		Antisense	TGCTGATGCCTTAGCAGTGG
Ir	XM006248753.2	Sense	GGAGGGTCCTCTCTGAGCAT
		Antisense	CCTGGAGGCAGTCAAACTACA
Slc2a1 (Glut1)	NM138827.1	Sense	CAGCCACTCTCCTATCTCCAC
		Antisense	TCCTGCGGTAAAGCCTCCTA
P53	NM030989.3	Sense	AGATAGTACTCGGCCCCCTC
		Antisense	TAGGTGCCAGGTCCAACAAC
Tbp	NM001004198.1	Sense	CTCAGTTACAGGTGGCAGCA
		Antisense	GCACAGAGCAAGCAACTCAC
Hprt	NM_012583.2	Sense	GCCTAAAAGACAGCGGCAAG
		Antisense	GGCTGCCTACAGGCTCATAG

Hk2	NM_012735.2	Sense	TGTCTCTGTATATTCACTGTAG
		Antisense	GCGAACAACFCCTTACTT
Pkm2	NM_053297.2	Sense	TGCTGAAGGAGATGATTA
		Antisense	ACGGACATTCTTGATAGT
Ldh-a	NM_017025.1	Sense	CGAGAGCATAATGAAGAAC
		Antisense	TCCTTGATTCCATAGAGAC
Cpt1a	NM_031559.2	Sense	TTACAAGGCAGAAGAGTG
		Antisense	GCAATGGACAGATTAGAAC
Fasn	NM_017332.1	Sense	TACCTTAACACGACTCAAC
		Antisense	TGATGGAACCTTCAATGG
Acaca	NM_022193.1	Sense	GTCACACTTCACTCTATG
		Antisense	CTGTCATCCTCAATATCG
Srebp1	NM_001276707.1	Sense	TACCGTTCCTCTATCAAT
		Antisense	AGACAGCAGATTTATTCAG
Hadha	NM_130826.2	Sense	CAGCACGAATATCACAAGAAGGA
		Antisense	TGATGGCGGCAACAACAG
Acads	NM_022512.2	Sense	ATGCCAACTCCTGGGCTTAC
		Antisense	GGGATGCGACAGTCCTCAAA
Pparγ	NM_013124	Sense	GCCTGCGGAAGCCCTTTGGT
		Antisense	CAGCAAGCCTGGGCGGTCTC

Table 2. Antibodies used for flow cytometry.

Marker	Antibody	Brand
CD3	Vio Blue conjugated anti-rat CD3	(Miltenyi Biotech, Germany)
CD4	PE-Vio770 conjugated anti-rat CD4	(Miltenyi Biotech, Germany)
CD8b	FITC conjugated anti-rat CD8b	(Miltenyi Biotech, Germany)
CD45	APC conjugated anti-rat CD45	(Miltenyi Biotech, Germany)
CD45R	PE Vio770 conjugated anti-rat CD45R	(Miltenyi Biotech, Germany)
CD161	PE conjugated anti-rat CD161	(Miltenyi Biotech, Germany)
CD43	APC-Vio770 conjugated anti-rat CD43	(Miltenyi Biotech, Germany)
