# Supplementary material for: Circadian disruption promotes tumor growth by anabolic host metabolism; experimental evidence in a rat model
Source: BMC Cancer. 2017 Sep 6;17:625. doi: 10.1186/s12885-017-3636-3 (PMC5585981; doi:10.1186/s12885-017-3636-3)
Supplement: Supplementary file 2 — Supplementary methods. (PDF 67 kb) [file 12885_2017_3636_MOESM2_ESM.pdf]

Additional file 2

Experimental Procedure

General housing conditions were the same as described in the Method section.

After baseline, rats were randomly assigned to one of 2 groups, which differed in experimental diet. 1. Chow diet rats (Chow, n=4) received *ad libitum* access to chow diet (Rodent Laboratory Chow 5001, Purina, Minnetanka, MN, USA) for 4 weeks. 2. High sugar diet rats (HS, n=7) received daily 1 h access to a high sugar diet (pellets were mixed with sugar) from ZT4 to ZT5 for 4 weeks. All rats were given free access to water. Body weight was determined at the beginning and every week along the protocol, chow and sugar diet consumption was assessed at the end of the 4 th week during 24h in order to calculate the Kcal ingestion per day. On week 4 a GTT was performed as described in the methods and sugar diet was interrupted and both groups remained under a chow diet. Before tumor cells inoculation blood samples were taken from the tail vein in order to measure glucose and TG plasma levels. Rats were subcutaneously inoculated with C6 tumor cells. After 13 days of tumor inoculation, rats were euthanized and tumors as well as blood were collected for further metabolic analysis of the host and tumor.
