# Supplementary figures and images for: Circadian disruption promotes tumor growth by anabolic host metabolism; experimental evidence in a rat model
Source: BMC Cancer. 2017 Sep 6;17:625. doi: 10.1186/s12885-017-3636-3 (PMC5585981; doi:10.1186/s12885-017-3636-3)

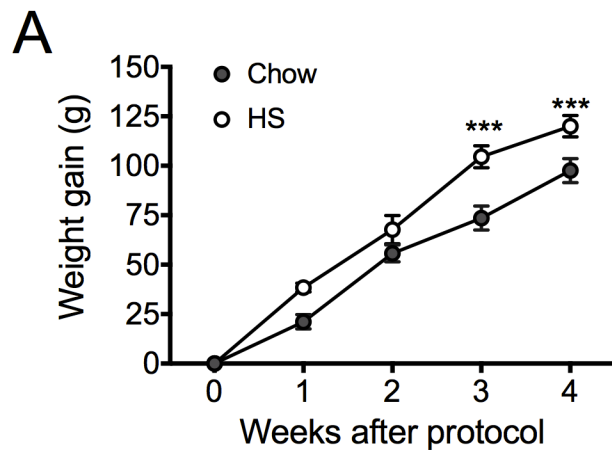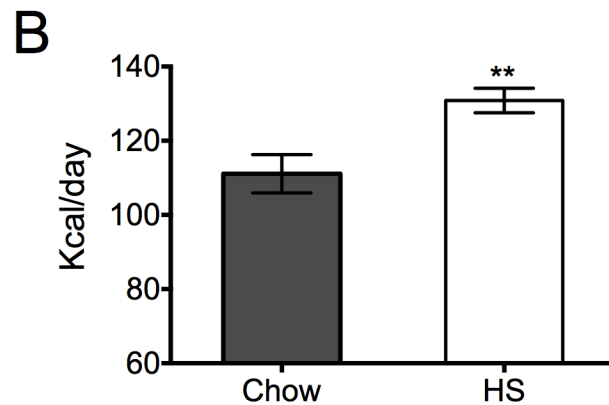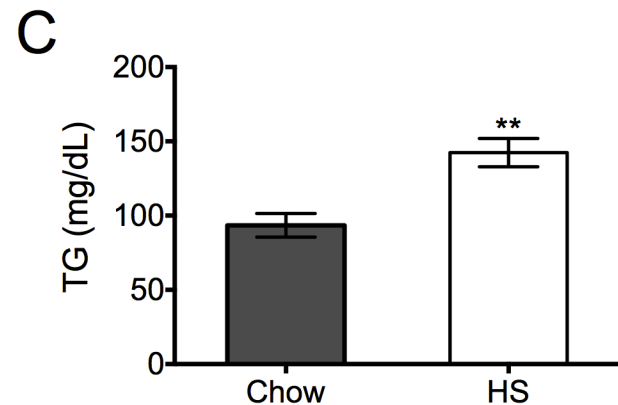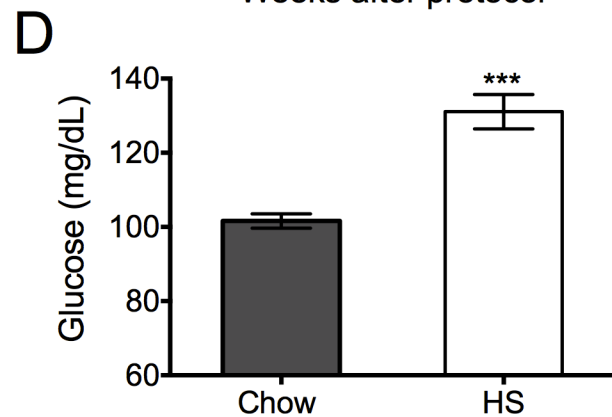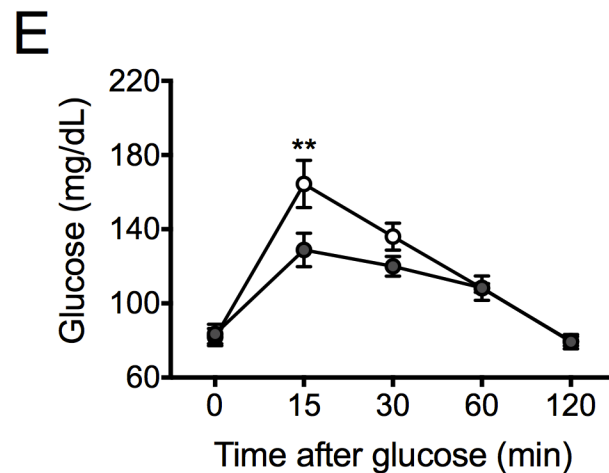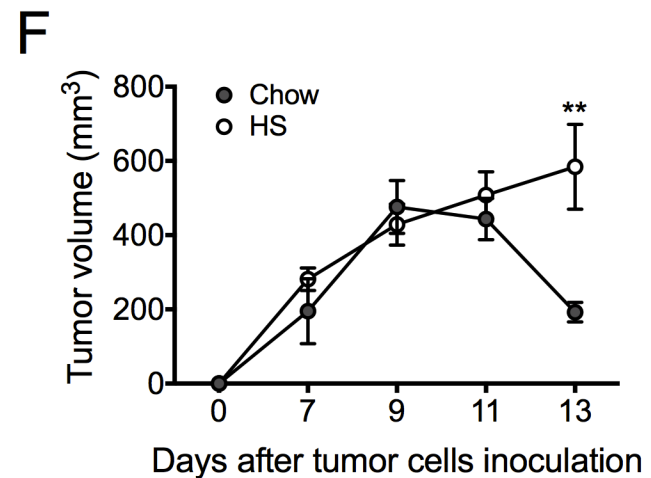

Supplement: Supplementary file 3 — High sugar diet (HS) induces a suitable metabolic environment for tumor growth. (A) HS rats (white circles) gained more body weight along the 4-week protocol as compared with chow diet rats (grey circles). Data are the mean ± SEM (n = 7/group). The repeated-measures two-way ANOVA indicated significant effects for condition versus time, interaction p = 0.0014. The Bonferroni test ***p < 0.001 indicated statistical difference from chow diet. (B) HS rats ingest more Kcal in 24 h. Data are the mean ± SEM (n = 7/group). ** p < 0.01 indicates statistical difference from chow diet; unpaired t test. (C) Basal plasma triglycerides (TG) and glucose levels (D) under ad libitum conditions were significantly increased in HS rats. Data are the mean ± SEM (n = 6–7/group), **p < 0.01, ***p < 0.001 indicates statistical difference from chow diet; unpaired t test. (E) Glucose tolerance test (GTT, 0–120 min) following i.p. administration of 1 g of glucose/kg. Values are expressed as mean ± SEM (n = 7/group). The repeated-measures two-way ANOVA indicated significant effects for condition versus time interaction p = 0.016. The Bonferroni test **p < 0.01 indicated statistical difference from chow diet. (F) Tumor volume along 13 days after subcutaneous C6 cells inoculation. The repeated-measures two-way ANOVA indicated a significant interaction for condition versus time, p = 0.0032. Data are expressed as mean ± SEM (n = 4-7group). The Bonferroni test indicated **p < 0.01 statistical difference from chow diet. (PDF 328 kb) [file 12885_2017_3636_MOESM3_ESM.pdf]

A

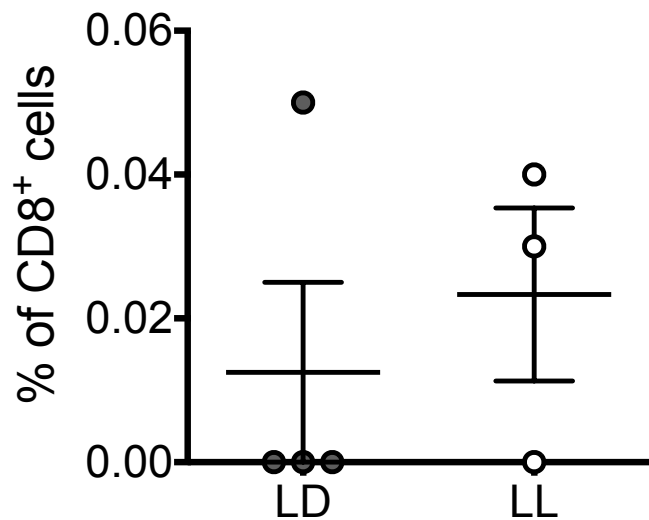

B

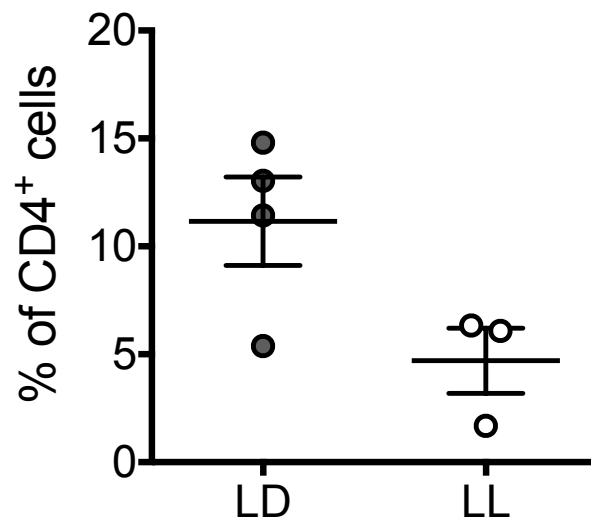

C

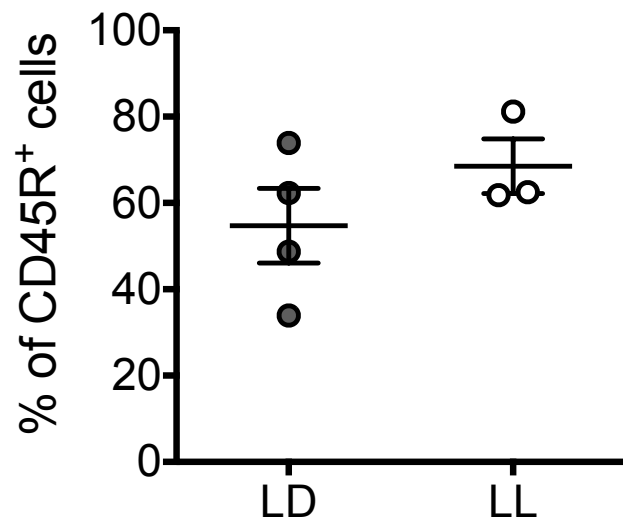

D

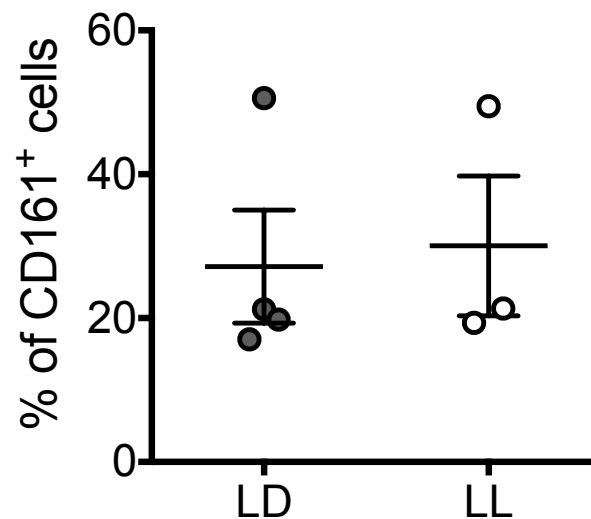

Supplement: Supplementary file 4 — Tumors from LL and LD rats similar percentages of immune cells. (A) Percentage of CD8+, (B) CD4+, (C) CD45R+ and (D) CD161+ cells. Data are expressed as the mean ± SEM (n = 3–4/group). Analysis was done on whole lysates from tumors removed from LD and LL rats on day 13. (PDF 23 kb) [file 12885_2017_3636_MOESM4_ESM.pdf]
